# Comparison of RetinaNet-Based Single-Target Cascading and Multi-Target Detection Models for Administrative Regions in Network Map Pictures

**DOI:** 10.3390/s22197594

**Published:** 2022-10-07

**Authors:** Kaixuan Du, Xianghong Che, Yong Wang, Jiping Liu, An Luo, Ruiyuan Ma, Shenghua Xu

**Affiliations:** 1 School of Resource and Environmental Science, Wuhan University, Wuhan 430079, China; 2 Research Center of Geospatial Big Data Application, Chinese Academy of Surveying and Mapping, Beijing 100830, China

**Keywords:** map pictures, RetinaNet, single-target cascading model, multi-target model, administrative regions

## Abstract

There is a critical need for detection of administrative regions through network map pictures in map censorship tasks, which can be implemented by target detection technology. However, on map images there tend to be numerous administrative regions overlaying map annotations and symbols, thus making it difficult to accurately detect each region. Using a RetinaNet-based target detection model integrating ResNet50 and a feature pyramid network (FPN), this study built a multi-target model and a single-target cascading model from three single-target models by taking Taiwan, Tibet, and the Chinese mainland as target examples. Two models were evaluated both in classification and localization accuracy to investigate their administrative region detection performance. The results show that the single-target cascading model was able to detect more administrative regions, with a higher f1_score of 0.86 and mAP of 0.85 compared to the multi-target model (0.56 and 0.52, respectively). Furthermore, location box size distribution from the single-target cascading model looks more similar to that of manually annotated box sizes, which signifies that the proposed cascading model is superior to the multi-target model. This study is promising in providing support for computer map reading and intelligent map censorship.

## 1. Introduction

Maps are manifestations of devising, summarizing, and modelling natural and social phenomena with a definite mathematical foundation and symbol system, and convey abundant geographic information [1]. They are of great significance in production, construction, planning, navigation, and daily travel. Furthermore, labels, indicators, and boundaries on maps represent national sovereignty and territorial integrity, thus making it crucial to produce and utilize correct maps [2]. However, there are currently numerous problematic maps on the internet, including incorrect national and regional administrative boundary depictions and missing isolated islands, which tends to result in national and regional disputes [3]. Therefore, it is imperative to quickly and accurately recognize these regions of interest on a map and then facilitate detection of incorrect regions.

The essence of recognizing regions of interest on the map is actually target detection in computer vision. Target detection aims at recognizing whether the target exists and where it is indicated by bounding boxes on the images. In the past, target detection normally relied on visual interpretation, which is inefficient and labor-intensive despite having the highest detection accuracy [4]. With the advancement of computer technology, most traditional target detection techniques developed hand-crafted (engineered) features derived from Sobel edge recognition [5], Haar-like detectors [6], histogram of oriented gradient (HOG) detectors [7], and deformable part-based models (DPMs) [8], etc., and then detect the target by utilizing developed features and shallow classifiers or regressors such as support vector machines (SVMs) [9] and boosting [10]. Although these methods are reliable and inexpensive, feature derivation is heavily dependent on hand-engineering, thus limiting the ability to make an inference with the extracted features in real time [11]. It is difficult to use these methods for quickly, robustly, and automatically detecting targets.

Recently, with large amounts of data available and improved computational power, deep convolutional neural networks [12] and their widespread applications in image classification [13] have emerged, and target detection techniques based on deep learning have achieved significant progress [14,15]. Compared to traditional techniques, deep learning techniques can automatically extract hierarchical feature vectors/representations from the underlying data and can be disentangled through multilevel nonlinear mapping [16]. There are two main paradigms for target detection based on deep learning: two-stage detectors such as R-CNN [17] and R-FCN [18] and one-stage detectors such as YOLO [19], SSD [20], and RetinaNet [21]. The process of two-stage detection can be split into two parts: proposing regions and classifying and regressing bounding boxes. One-stage detectors directly map class predictions of different objects present at each location of the extracted feature maps without utilizing the region classification step. Two-stage detectors tend to achieve good detection performance but have decreased overall detection speed due to generating region proposals. Although one-stage detectors are sometimes not able to achieve as good a performance as the two-stage, they are comparatively more time-efficient and hence are greatly applicable for real-time target detection [22,23]. One-stage detectors have been largely applied under specific application scenarios, such as face detection [24,25], text detection [26,27], vehicle detection [28], traffic sign detection [29], pedestrian detection [30,31], and remote sensing target detection [32,33], but to our knowledge, they have rarely been applied to administrative region detection on a map to facilitate problematic map identification. Furthermore, it is still underexplored whether better performance could be achieved by using a deep learning model to detect multiple classes of targets simultaneously rather than only one specific class. Considering the current state-of-the-art target detector of RetinaNet by implementing a focal loss cross-entropy function and feature pyramids network (FPN) [21,34,35], the primary contributions of this study were to (1) deploy the RetinaNet model to detect multiple administrative regions on maps; and (2) evaluate the performance between simultaneous detection of multiple targets and cascading detection of single targets.

## 2. Method

### 2.1. RetinaNet Model Structure

**Figure 1:**
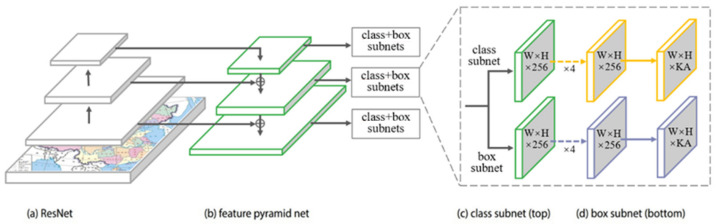
Basic network model structure of RetinaNet.

The RetinaNet model is composed of two backbone networks for calculating the convolutional feature map of the input image [21], i.e., ResNet50 and FPN, and two task-specific subnetworks used for classification and bounding box regression (Figure 1). RetinaNet adopts ResNet50 to avoid gradient vanishing in extracting more features with the increasing convolutional layers, because it has an additional identity mapping capability. Subsequently, RetinaNet adopts the feature pyramid network (FPN) [34] as its backbone, which is in turn built on top of ResNet50 in a fully convolutional fashion. The fully convolutional nature enables the network to take an image of an arbitrary size and output proportionally sized feature maps at multiple levels in the feature pyramid. The FPN enhances extracted features from the convolutional network through the top-down method and horizontal connections by transforming the network from the input image to a multiscale feature pyramid, which enables the model to detect administrative regions of various sizes and increases the speed. At the same time, the area of the anchor box increases from 32 × 32 to 512 × 512, and three scales (20, 21/3, and 22/3) and three aspect ratios (1:2, 1:1, and 2:1) are used in each feature pyramid. Therefore, the feature points of each layer feature map correspond to 9 anchor boxes, which improves the average precision to a certain extent. Based on the multiscale feature pyramid, two subnetworks of classification and box regression were constructed through simple convolution operations. Specifically, the classification subnetwork predicts the probability of each anchor box class, which is a fully convolutional network (FCN) attached to each FPN level and shares parameters. The subnet consists of four 3 × 3 convolutional layers with 256 filters, followed by ReLU activations. Then, another 3 × 3 convolutional layer with K × A filters is connected, where K is the number of categories of detection targets, and A is the number of anchor boxes. The category is predicted at each position through sigmoid activation (Figure 1). Similarly, the regression subnet is attached to each feature map of the FPN in parallel to the classification subnet. The design of the regression subnet is identical to that of the classification subnet, except that the last convolutional layer is 3 × 3 with 4 × A filters (Figure 1).

### 2.2. Focal Loss

RetinaNet introduces a new loss function, focal loss (FL). Focal loss is an improved CE (cross-entropy) loss function. The model with focal loss reduces the contribution of negative samples in model training by passing a weakening index on the basis of the original CE loss. Consequently, it solves the problem of unbalanced positive and negative samples in the training of the target detection model and improves model accuracy [21]. The loss in RetinaNet is a multi-task loss of classification (hereafter namely $L_{cls}$) and regression (hereafter namely $L_{reg}$), which can be written as:

(1) $$L=\lambda L_{reg}+L_{cls},$$

In Equation (1), λ is a hyperparameter that controls the balance between the two task losses. The classification loss $L_{cls}$ for each anchor is expressed as follows:

(2) $$L_{cls}=-\sum_{i=1}^{K}(y_{i}\log\left(p_{i}\right)\left(1-p_{i}{)}^{\gamma }\alpha_{i}+\left(1-y_{i}\right)\log\left(1-p_{i}\right)p_{i}^{\gamma }\left(1-\alpha_{i}\right)\right),$$

where *K* denotes the number of classes; $y_{i}$ equals 1 if the groundtruth belongs to the i-th class and 0 otherwise; $p_{i}$ is the predicted probability for the i-th class; *γ* ∈ (0, +∞) is a focusing parameter; and$\;\alpha_{i}$ ∈ [0, 1] is a weighting parameter for the i-th class. In reality, negative samples that are matched to the target in an image account for a large portion of inputs, which can be easily classified by the detector as background and contribute no useful learning signal, overwhelming the loss and computed gradients and leading to degenerated models. The focal loss function introduces the focusing parameter *γ* to downweight the loss assigned to easily classified examples. This effect increases as the value of γ increases and makes the network focus more on positive samples. The balancing parameter α is also useful for addressing class imbalance.

The regression loss $L_{reg}$ represents $smooth_{L1}$ loss of the bounding box regression [21], and can be defined as:

(3) $$L_{reg}=\sum_{j\in \left\{x,y,w,h\right\}}smooth_{L1}\left(P_{j}^{i}-T_{j}^{i}\right),$$

Here, for each anchor A with a match, the regression subnet predicts four numbers as Pi = ($P_{x}^{i}$, $P_{y}^{i}$, $P_{w}^{i}$, $P_{h}^{i}$), which represent the center coordinates, width, and height of the anchor bounding box. A regression target *Ti* is computed as the offset between the anchor A and the groundtruth G as follows:

(4) $$T_{x}^{i}=\frac{\left(G_{x}^{i}-A_{x}^{i}\right)}{A_{w}^{i}},$$

(5) $$T_{y}^{i}=\frac{\left(G_{y}^{i}-A_{y}^{i}\right)}{A_{h}^{i}},$$

(6) $$T_{w}^{i}=\log\left(\frac{G_{w}^{i}}{\;A_{w}^{i}}\right),$$

(7) $$T_{h}^{i}=\log\left(\frac{G_{h}^{i}}{\;A_{h}^{i}}\right),$$

where ${smooth}_{L1}\left(x\right)$ is *smooth* *L1* loss, which can be defined as:

(8) $${Smooth}_{L1}\left(x\right)=\left\{\begin{array}{c} 0.5x^{2}\;\;\;\;\;\;\;\;\left|x\right|<1\\ \left|x\right|-0.5\;\;\left|x\right|\geq 1 \end{array}\right.,$$

### 2.3. Single-Target Cascading and Multi-Target Detection Using RetinaNet Models

Considering the different sizes and shapes of administrative regions on the maps, this study designed two strategies using the RetinaNet architecture (hereafter referred to as single-target cascading detection and multi-target detection) to explore their performance for detecting administrative regions on the maps. Specifically, in the single-target cascading detection model, the RetinaNet model was trained for each class of targets separately. That is to say, each class of targets had a specific model. For detection, a map goes through these models one by one, and the detected results are aggregated by the union operator to derive the final detection result. The principle of target extraction by the single-target cascading detection model is shown in Equation (9).

(9) $$F_{M}\left(x\right)=\cup_{m=1}^{n}h\left(x;a_{m}\right),$$

where $h\left(x;a_{m}\right)$ is a single-target detection model, $a_{m}$ is parameters in the model, and *x* is the input image. $F_{M}\left(x;P\right)$ is the final target detection model, and n is the number of target classes.

**Figure 2:**
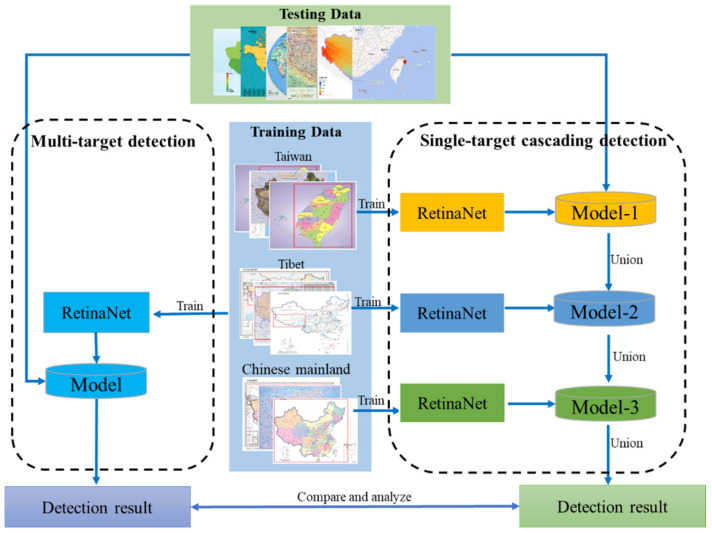
Single-target cascading and multi-target detection model.

In contrast, the multi-target detection strategy takes all classes of targets as the input for one RetinaNet model and generates a trained model for simultaneously detecting multiple class targets. The specific structure of the two strategies is shown in Figure 2.

## 3. Experiments

In this section, we introduce the dataset and the evaluation indexes used in our experiment and then illustrate the detailed information of the experiment.

### 3.1. Dataset

**Figure 3:**
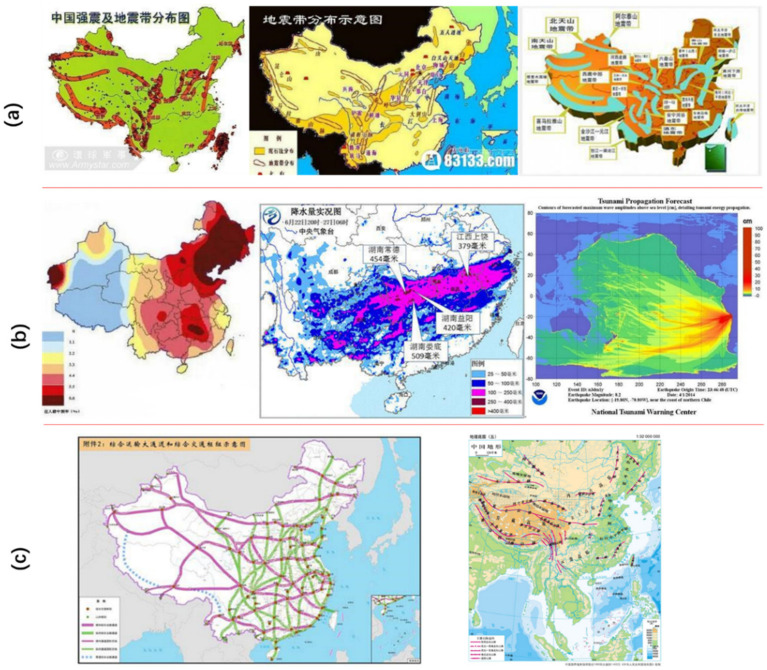
Different thematic maps: (**a**) seismic zone; (**b**) climate; (**c**) transportation and mountain terrain.

Three administrative regions (targets) of the map are used in this paper, which include Taiwan province, Tibet province, and the Chinese mainland in China. Using an orientation crawler approach, 3991 map images were derived from an internet image search engine using the keywords Taiwan, Tibet, Chinese mainland, and map. We randomly divided 3192 images into a training set and 799 images into a test set. Map images from the internet have a broad range of image size. Both large and small target regions were included in the sample dataset. The size of Taiwan varied from 24 pixels × 14 pixels to 3240 pixels × 1819 pixels. The size of Tibet varied from 24 pixels × 14 pixels to 3240 pixels × 1819 pixels, and the Chinese mainland varied from 32 pixels × 21 pixels to 2496 pixels × 2863 pixels. We split samples with different sizes into the training and test datasets randomly. Furthermore, the datasets in this study contained different kinds of thematic maps, such as seismic zone maps, climate maps, transportation maps, and terrain mountain maps, etc., as shown in Figure 3.

The test set was intended to provide an unbiased evaluation of a trained model using the training set. The specific distribution is shown in Table 1. The three targets in the map images were manually annotated using a web-based image annotation tool. The tool outputs an annotation file with an interactive drawing of a bounding box containing all the pixels of the target, which include the directory of each image, the coordinates of the top left corner for the annotated bounding box, the width and height of the annotated bounding box, and the name of the target (Table 2). The principle of manual annotation is to use the smallest possible box to completely cover the targets but get rid of the useless background.

### 3.2. Evaluation Metrics

We used four evaluation metrics, including intersection over union (*IOU*), precision, recall, and harmonic mean of precision and recall [36,37]. *IOU* is used to measure how much our predicted boundary overlapped with the ground truth (the target’s real boundary), which calculated the coincidence degree between the predicted box and the ground truth box. *IOU* is defined by Equation (10), where $B_{p}$ represents the predicted bounding box and $B_{gt}$ represents the ground truth bounding box. The threshold of *IOU* indicates whether the detection is valid or not.

(10) IOU=areaBgt∩BpareaBgt∪Bp,

Here, if the predicted bounding box overlapped with the annotated bounding box and exceeded the *IOU* threshold (i.e., 0.5 in classifying whether the prediction was a true positive or a false positive), the predicted bounding box represented the administrative region sample; otherwise, it was the background sample.

Precision and recall represent the classification accuracy of the model, where precision measures the accuracy of detected targets and recall measures the integrity of detection. An *f1* score is a comprehensive evaluation metric that measures the accuracy of a classification model by calculating the average of precision and recall. The three metrics can be calculated as follows:

(11) $$precision=\frac{TP}{TP+FP},$$

(12) $$recall=\frac{TP}{TP+FN},$$

(13) $$f1_{score}=\frac{2*precision*recall}{precision+recal},$$

where *TP* denotes the number of correctly predicted administrative region samples; *FN* denotes the number of wrongly classified samples; and *FP* denotes the number of incorrectly identified samples.

Average precision (*AP*) balances the precision (*P*) and recall (*R*) values, reflecting the performance of the model in each class, which is the area under the precision-recall curve, as shown in Equation (14) [38]. The *mAP* is the average of *AP* for target classes, which is used to show the model’s advantages and disadvantages across all classes (Equation (15)). This study used the value of *mAP* when the *IOU* threshold = 0.5 [38,39].

(14) $$AP\left(n\right)=\int_{0}^{1}p\left(r_{n}\right)dr_{n},$$

(15) $$mAP=1/n\sum_{1}^{n}AP\left(n\right),$$

### 3.3. Implementation Details

Model training was carried out on the Ubuntu18.04.6 LTS operating system. The computer hardware configuration was as follows: Intel (R) Core (TM) i9-10900X, 64 G, NVIDIA GeForce RTX 3090, and GPU with 24 G memory. The model training environment was Python 3.6 and Keras 2.4.3. The ResNet50 backbone network was initialized with ImageNet training set parameters. The initial learning rate was 0.0001 during the training process. A total of 100 epochs were trained, and the batch was set to one image. Weight attenuation was set to 0.0001 and momentum was set to 0.9. *γ* was set to 2, and *α* was set to 0.25 in the focal loss function.

### 3.4. Experimental Results

**Figure 4:**
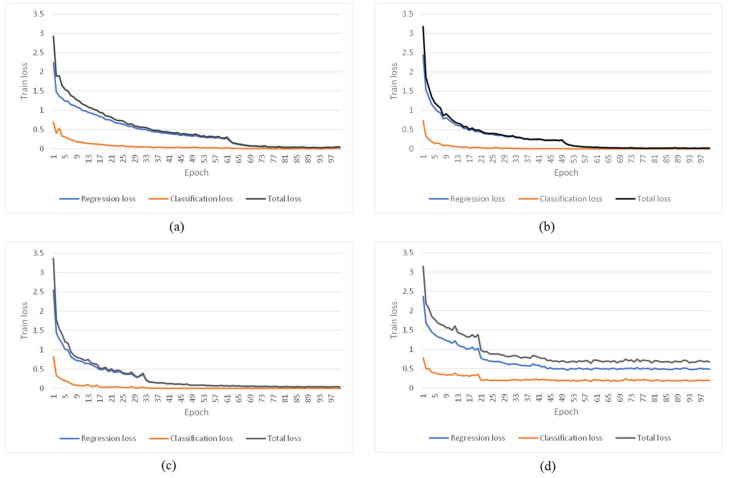
Training loss of single-target and multi-target detection models: (**a**) Training loss of model for target 1 (Taiwan); (**b**) Training loss of model for target 2 (Tibet); (**c**) Training loss of model for target 3 (Chinese mainland); (**d**) Training loss of model for all three targets.

Figure 4a–c shows the total loss, classification loss, and regression loss curves of three training models for three targets, respectively, and Figure 4d shows the training model with all three targets. It can be seen that the loss curves of both the single-target and multi-target detection models flatten out, and the models tend to converge after 100 epochs of training processing. However, both the classification loss and regression loss of the single-target model is lower than the multiple target training model, which demonstrates the single-target training model performs better. In addition, the classification losses are always lower than the regression losses for all training models. Although the training loss of the multi-target detection model are higher than the single-target model, the multi-target detection model needed fewer epochs—about 40—to get a relatively stable regression loss value. The training duration of three single-target detection models was 24,546 s, 7303 s, and 5211 s, respectively. The training duration of the multi-target detection model was 32,692 s.

The single-target and multi-target trained detection models were also evaluated with the testing samples, as shown in Table 3 and Table 4, respectively. The detection results from single-target cascading detection models have higher *P*, *R*, and *f1_score* values (0.80, 0.93 and 0.86, respectively) than the multi-target model (0.77, 0.44, and 0.56, respectively), which indicates that the single-target cascading model is superior to the multi-target model. Moreover, the multi-target model has apparent omission errors, with an *R* of 0.44, especially for the Chinese mainland (0.05).

**Figure 5:**
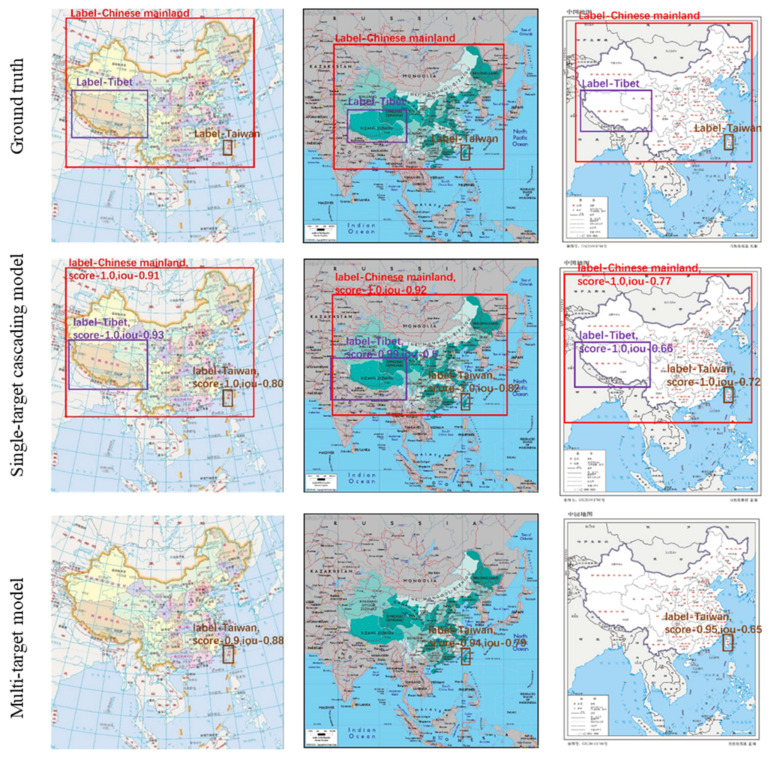
Annotations of different targets in maps (**top row**) and detected results from the single-target cascading model (**middle row**) and multi-target model (**bottom row**). (Different colors of the boxes indicate different types of targets, and labels indicate the target types; score is for classification confidence; *IOU* is for localization confidence).

The results of three administrative regions detected by single-target cascading and multi-target models were visually compared with the manual annotations (ground truth), and some examples are shown in Figure 5. The rectangular frames are manually annotated, while the frames in the last two rows represent their prediction results from the single-target cascading and multi-target models, respectively. The recognized regions are marked by different rectangular boxes, highlighting the different administrative regions in the maps. It is clear that the single-target cascading model correctly detected three administrative regions while the multi-target detection model only detected the Taiwan target. A large region such as the Chinese mainland was difficult for the multi-target detection model to detect under the influence of a more complicated background compared to the other two targets, which is consistent with the quantitative results of Table 3 and Table 4.

**Figure 6:**
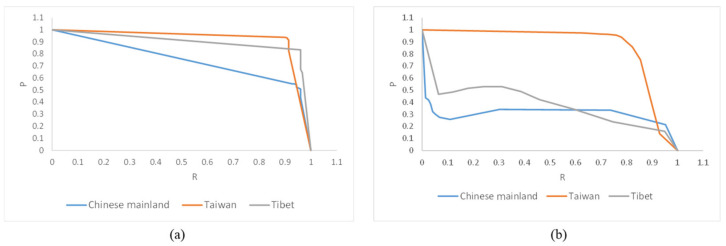
P–R curves of two categories of models: (**a**) single-target cascading model; (**b**) multi-target model.

Figure 6 shows the P–R curves of the Taiwan, Tibet, and Chinese mainland regions detected by the single-target cascading and multi-target models. Obviously, Taiwan has the highest precision for two categories of model among three administrative regions. The single-target cascading model was able to maintain a higher precision when having the same recall compared to the multi-target model, especially for Tibet and the Chinese mainland. The precision of the multi-target mode rapidly decreased with the increasing recall, but the single-target cascading model could better balance precision and recall.

**Figure 7:**
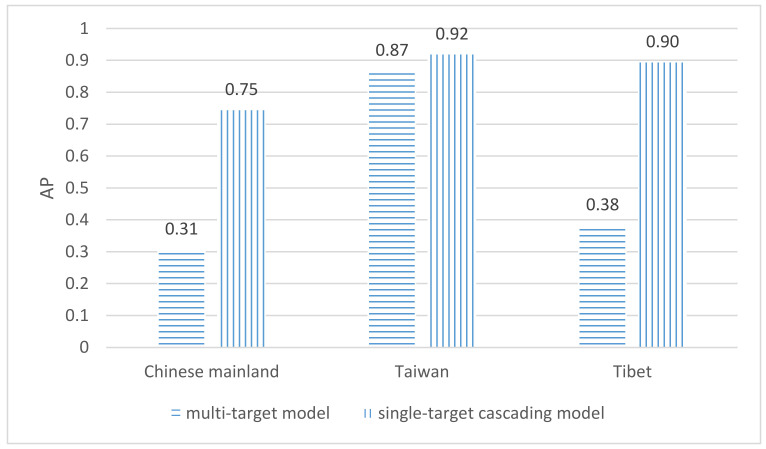
Comparison of AP between the single-target cascading model and the multi-target model.

The *AP* values for each administrative region are also compared in Figure 7. All *AP* values of the three administrative regions from the single-target cascading model were higher than the multi-target model, especially for Tibet. The mean *AP* (*mAP*) is 0.85 and 0.52 for the single-target cascading and multi-target detection models, respectively. This also implies the superiority of the single-target cascading detection model in detecting regions of interest on maps.

**Figure 8:**
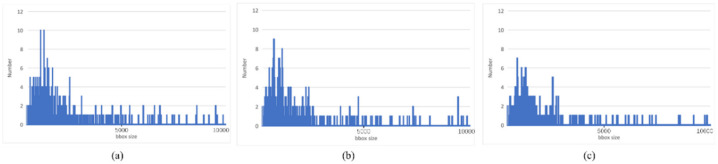
Distribution of ground truth and prediction: (**a**) Distribution of box size in ground truth; (**b**) Distribution of box size in prediction of single-target cascading model; (**c**) Distribution of box size in prediction of multi-target model.

As shown in Figure 8, the ground truth and predicted sizes from the single-target cascading model have more similar distributions than the multi-target model. Box size was primarily distributed in the range of (0, 10,000), with the largest number in the range of (500, 1000). This comparison demonstrates the single-target cascading model had more precise location regression results.

## 4. Discussion

With the advances in computer vision technologies and the overwhelming availability of open-source big data, deep learning-based target detection has become a popular research topic. However, previous studies primarily focused on entity targets such as faces, pedestrians, vehicles, traffic signs, text, roads, etc. [24,25,26,27,28,29,30,31,32,33]. These targets normally have specific colors, textures, or shapes. More importantly, these targets tend to be visually obvious and uncovered by other elements, which enable a deep learning-based model to easily extract features for detection. In contrast, unlike traditional maps created by professional cartographers in the past, current map resources vary greatly because of free creation, publishing, editing, and sharing on the Internet. This inevitably leads to different cartographic semantics, data descriptions, drawing standards, and design patterns on the administrative regions of maps as well as many covered elements on the administrative regions, which complicate the desired region detection [35,40,41,42,43]. Although there exist some studies to detect cartographic semantics using deep learning techniques, most of them focused on detecting map text and symbols using convolutional neural networks (CNNs) and generative adversarial networks (GANs) [44,45,46,47,48,49]. It has rarely been explored how to intelligently detect which administrative regions are included in a map. This study built region-detection models with high accuracy to detect the Chinese mainland, Taiwan, and Tibet, which is capable of migration, and can be extended to any administrative region.

This study proposed a RetinaNet-based cascading model to detect Chinese mainland, Taiwan, and Tibet maps and presented a series of experiments to evaluate its performance. The results show that the model obtains higher mAP for three regions in the map. We used the loss function and FPN to strengthen the learning of administrative region features, which further advanced the utilization rate of features of regions of interest and inhibited feature learning on the background or adjacent regions to improve the detection effect for regions of interest and to accelerate the convergence speed of the final results. From the test results of this model, it can be concluded that this model has a good auxiliary effect on administrative region screening and has certain application prospects, such as intelligent recognition of problematic maps and map-to-text conversion.

Meanwhile, we evaluated the performance of single-target cascading and multi-target detection with RetinaNet-based models, which indicated the superiority of single-target cascading detection. Multi-target training is a more complex learning process than the single-target one. That is to say, the feature learning for multiple targets requires more parameters to train the model. Using the same training data, it is easier to achieve a single-target detection. The process of cascaded single-target models for detection is similar to the process of constructing a piecewise function where AdaBoost theory is employed to combine multiple classifiers to obtain a robust classifier [50,51].

There are also some limitations for the proposed RetinaNet-based single-target cascading model. For example, the Chinese mainland region had the lowest accuracy, and this is mainly because the Chinese mainland region has a more complex background compared to the Taiwan and Tibet regions. FPN was adopted to increase the richness of features represented by each pyramid ladder in the pyramid-shaped model, using a multiscale feature extracted from the ResNet50 layer, but the omission error occurred for Chinese mainland region. Some improved strategies, such as adding a multiple-level feature graph [34], adopting adaptive training sample selection [49], and designing a loss weight adjustment [52] can be investigated to enhance the target training for the proposed model in the future.

## 5. Conclusions

In this paper, taking Taiwan, Tibet, and the Chinese mainland as target examples, a single-target cascading model was constructed by training three single-target models based on the widely-used RetinaNet model. At the same time, a multi-target model was built with all three target samples. Their comparisons for classification and location evaluation shows the RetinaNet-based single-target cascading model can better detect administrative regions on the network map pictures. This model also can be extended to any other administrative region. This study will greatly reduce the workload for map review specialists and improve their work efficiency.

## Figures and Tables

**Table 1:** Sample distribution of different targets.

	Region of Interest	Training Dataset	Test Dataset	Total
Target 1	Taiwan	2151	538	2689
Target 2	Tibet	582	146	728
Target 3	Chinese mainland	459	115	574
Total		3192	799	3991

**Table 2:** Target annotation format.

No.	path_img_file	box_x	box_y	Width	Height	Label
1	dataset/image_0001.jpg	890	659	944	743	Taiwan
2	dataset/ image_0002.jpg	775	631	845	721	Taiwan
3	dataset/ image_0003.jpg	36	57	762	535	Xizang
4	dataset/ image_0004.jpg	5	51	536	316	Xizang
5	dataset/ image_0005.jpg	5	2	341	289	Chinese mainland
6	dataset/ image_0006.jpg	95	93	666	546	Chinese mainland

**Table 3:** Accuracy statistics of different targets with the single-target cascading detection model.

	Single-Target Model		Single-Target Cascading Detection Models	
	Taiwan	Tibet	Chinese Mainland	Taiwan, Tibet, and Chinese Mainland
Precision (P)	0.92	0.77	0.52	0.80
Recall (R)	0.91	0.96	0.94	0.93
f1_socre	0.92	0. 86	0.67	0.86

**Table 4:** Accuracy statistics of different targets with the multi-target detection model.

	Multi-Target Model			
	Taiwan	Tibet	Chinese Mainland	Taiwan, Tibet, and Chinese Mainland
Precision (P)	0.94	0.53	0.3	0.77
Recall (R)	0.78	0.31	0.05	0.44
f1_socre	0.85	0.39	0.10	0.56

## Data Availability

Not applicable.
